# Thrombotic microangiopathies after kidney transplantation in modern era: nosology based on chronology

**DOI:** 10.1186/s12882-023-03326-8

**Published:** 2023-09-20

**Authors:** Florent Von Tokarski, Alexandre Fillon, Valentin Maisons, Benjamin Thoreau, Guillaume Bayer, Philippe Gatault, Hélène Longuet, Bénédicte Sautenet, Matthias Buchler, Cécile Vigneau, Fadi Fakhouri, Jean-Michel Halimi

**Affiliations:** 1 Service de Néphrologie-HTA, Dialyses, Transplantation Rénale, Hôpital Bretonneau Et Hôpital Clôcheville, CHU Tours, 2 Bd Tonnellé, 37044 Tours, Tours Cedex France; 2 EA4245, François-Rabelais University, Tours, France; 3 Inserm U1246, Hôpital Bretonneau, CHU Tours, Tours, France; 4 Service de Néphrologie, CHU Pontchaillou, 35033 Rennes, France; 5 Université Rennes 1, Inserm IRSET, UMR 1085, 35033 Rennes, France; 6 Department of medicine, Service of Nephrology, CHUV and Université de Lausanne, Lausanne, Switzerland

**Keywords:** Kidney transplantation, Malignant hypertension, Hemolytic uremic syndrome, Infection, Outcomes

## Abstract

**Background:**

Thrombotic microangiopathies (TMAs) are rare but can be severe in kidney transplant.

recipients (KTR).

**Methods:**

We analysed the epidemiology of adjudicated TMA in consecutive KTR during the.

2009–2021 period.

**Results:**

TMA was found in 77/1644 (4.7%) KTR. Early TMA (*n* = 24/77 (31.2%); 1.5% of all KTR) occurred during the first two weeks ((median, IQR) 3 [1–8] days). Triggers included acute antibody-mediated rejection (ABMR, *n* = 4) and bacterial infections (*n* = 6). Graft survival (GS) was 100% and recurrence rate (RR) was 8%. Unexpected TMA (*n* = 31/77 (40.2%); 1.5/1000 patient-years) occurred anytime during follow-up (3.0 (0.5–6.2) years). Triggers included infections (EBV/CMV: *n* = 10; bacterial: *n* = 6) and chronic active ABMR (*n* = 5). GS was 81% and RR was 16%. Graft-failure associated TMA (*n* = 22/77 (28.6%); 2.2% of graft losses) occurred after 8.8 (4.9–15.5) years). Triggers included acute (*n* = 4) or chronic active (*n* = 14) ABMR, infections (viral: *n* = 6; bacterial: *n* = 5) and cancer (*n* = 6). 15 patients underwent transplantectomy. RR was 27%. Atypical (*n* = 6) and typical (*n* = 2) haemolytic and uremic syndrome, and isolated CNI toxicity (*n* = 4) were rare. Two-third of biopsies presented TMA features.

**Conclusions:**

TMA are mostly due to ABMR and infections; causes of TMA are frequently combined. Management often is heterogenous. Our nosology based on TMA timing identifies situations with distinct incidence, causes and prognosis.

**Supplementary Information:**

The online version contains supplementary material available at 10.1186/s12882-023-03326-8.

## Introduction

Thrombotic microangiopathies (TMA) constitute a group of rare but deadly conditions after kidney transplantation. TMAs represent a major diagnostic and therapeutic challenge. TMAs are characterized by thrombocytopenia with hemolytic anemia, brain and renal involvement. Histopathology shows fibrin thrombi within glomeruli, in subendothelial areas and in the mesangium, associated with mesangiolysis, endothelial swelling, and corrugation of the glomerular basement membrane [1]. In the general population, TMA are separated into 2 groups of cause: primary (atypical hemolytic and uremic syndrome (aHUS) [2] and thrombotic thrombocytopenic purpura (TTP)) and secondary TMA that represent 94% of cases in the general population [3].

The incidence of TMAs in kidney transplant recipients (KTR) is unknown. A paper published 20 years ago derived from administrative codes indicated that the incidence of TMA code was 5.6 per 1000 person-year [4]. Most cited causes of TMA in KTR are calcineurin inhibitors (CNI) toxicity [5–8], atypical or typical HUS and mammalian target of rapamycin (mTOR) inhibitors’ toxicity [9]. Original data in this population are scarce [10, 11]. Presently, a long list of TMA causes (sometimes classified in “de novo” or “recurrent” TMA) are proposed, but it fails to provide a practical analysis of TMA in this population [5–8].

The objective of our study was to describe the epidemiology of TMA in consecutive KTR from our transplantation center to provide real-life causes, management, and outcome. We also proposed a new nosology based on the timing of TMA after transplantation.

## Patients and methods

### Selection of patients

KTR aged 18 years old or older who were admitted in our four-hospital institution (Centre Hospitalier Universitaire de Tours, Tours, France) between January 1, 2009, and December 31, 2021, with a suspicion of a first episode of TMA after kidney transplantation were included in this retrospective study.

### Adjudication of TMA cases

Data was individually collected by six physicians analysing patients’ hospitalization discharge summaries and allograft biopsy reports. The diagnosis of TMA was suspected in patients with at least three of the following parameters: hemoglobin < 120 g/L, increased lactate dehydrogenase (LDH), decreased haptoglobin, schistocytosis ≥ 0.5%, platelet count < 150,000/µL and biopsy features of TMA. Alternative diagnosis to TMA were excluded. TMA diagnostic and causes were then adjudicated by three physicians (C.V., F.F. and J.-M.H.) [3].

### Data collection

The following data were collected: primary renal disease, history of TMA, preformed and de novo donor specific antibodies (DSA) defined by a mean fluorescence intensity (MFI) ≥ 500 using Luminex® single-antigen bead assay, history of antibody-mediated rejection (ABMR) or T-cell mediated rejection (TCMR), donor’s age, cytomegalovirus (CMV) and Epstein-Barr virus (EBV) status, blood pressure, induction and maintenance immunosuppressive therapies and its serum trough levels, serum creatinine levels and allograft biopsy when available. All patients with a diagnosis of ABMR had a kidney biopsy.

Therapeutic management included change in maintenance immunosuppressive therapy (defined by discontinuation or lowering CNI of mTOR inhibitors dose), plasma exchange and specific therapeutics according to the cause of TMA. CNI toxicity was defined by tacrolimus trough level above 12 ng/ml.

### Outcomes during hospitalization and during follow-up

Outcomes included in-hospital adverse event such as death, major cardiovascular event (MACE, *i.e.,* nonfatal stroke, nonfatal myocardial infarction or cardiovascular death), renal replacement therapy (RRT) and allograft loss (defined as maintenance RRT). Outcomes at last follow-up included death, graft function (defined by serum creatinine levels) and end-stage renal failure (chronic dialysis or new transplantation), new onset ABMR or/and TCMR and recurrence of TMA.

### Statistical analysis

Continuous variables were presented as median (Interquartile range [IQR]). Non-parametric Mann–Whitney-Wilcoxon test or Kruskal–Wallis test followed by Dunn’s test were used for comparisons. Categorical variables were presented as numbers and percentage, and we used χ^2^-test or Fisher test for univariate analysis.

**Fig. 1:**
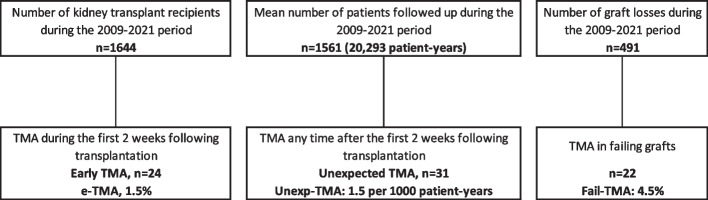
TMA in kidney transplant recipients

After a preliminary analysis of the timing of TMA during post-transplant management, it appeared that 3 distinct situations could be observed. The first group was constituted by patients who developed TMA within the first 2 weeks after transplantation early (early TMA: eTMA). The second group was constituted by the unexpected TMA anytime during follow-up, starting after the second week following transplantation (Unexp-TMA). The third group was constituted of patients with failing graft [12] who presented with TMA (Fail-TMA) (Fig. 1). For graft survival, only early and unexpected TMA groups were considered. Statistical analysis was conducted using GraphPad (version 8, Prism, San Diego.United State). The study was approved by the regional ethic committee (“Espace de reflexion éthique region Centre”: research project no. 2017–003).

## Results

### Baseline characteristics and incidence of TMA

During the 2009–2021 period, 1644 patients had a kidney transplantation whereas 2925 patients who received a transplantation since 1985 were followed up in our center (total follow-up period: 20,293 patient-years (/1,000-PY)). During this period, graft loss occurred in 491 patients (renal replacement therapy: *n* = 472; pre-emptive retransplantation: *n* = 19), whereas 493 patients died, and 14 patients were lost to follow-up. Overall, TMA occurred in 77 patients. We separated patients with TMA into 3 groups according to the timing of TMA during post-transplant management.

The first group of patients had a TMA during the first 2 weeks after transplantation (early-TMA group) representing 31.2% of all TMAs and 1.5% of all KTR. In this group, median time to TMA was 3 [IQR: 1–8]) days. Overall, 30% of eTMA patients had a previous transplantation, with frequent class II HLA antibodies and donor-specific antibodies (DSA); however, only 6 patients had DSA at the time of transplantation (Table 1). Thymoglobulin was used in 79% of patients. Median tacrolimus trough level at TMA diagnosis was in therapeutic range (8.7 ng/mL [5.1–11.5]).

**Table 1:** Characteristics of the population according to timing of TMA during post-transplant management

**Transplantation characteristics**	All	Early TMA	Unexpected TMA	Failure TMA	*P*-value
	*n* = *77*	*n* = 24	*n* = 31	*n* = 22	
**At the time of allograft transplantation**					
Previous transplantation, *n (%)*	23 (30)	9 (38)	7 (23)	7 (32)	0.46
Primary renal disease Glomerulonephritis, *n (%)*	28 (36) 5 (6) 7 (9) 6 (8) 6 (8) 6 (8) 17 (22)	8 (33)	13 (42)	7 (32)	0.72
Vascular/hypertension, *n (%)*		3 (13)	0 (0)	2 (9)	0.11
Obstructive uropathy/reflux nephropathy, *n (%)*		2 (5)	1 (3)	4 (18)	0.19
Diabetic nephropathy, *n (%)*		1 (4)	4 (13)	1 (5)	0.44
Interstitial nephritis, *n (%)*		2 (8)	3 (10)	1 (5)	0.88
Polycystic kidneys, *n (%)*		2 (8)	2 (6)	2 (9)	0.99
Unknown, *n (%)*		6 (25)	8 (26)	5 (23)	0.93
Anti-HLA class I positivity, *n (%)*	31 (40)	12 (50)	12 (39)	7 (32)	0.44
Anti-HLA class II positivity, *n (%)*	29 (38)	16 (67)	11 (35)	2 (9)	< *0.001*
Preformed anti-HLA DSA, *n (%)*	11 (14)	6 (25)	4 (13)	1 (5)	0.10
Donor age, year, *median (IQR)*	52 (42–66)	52 (38–72)	58 (46–76)	43 (29–57)	< *0.05*
Living donor, *n (%)*	4 (5)	2 (8)	2 (6)	0 (0)	0.55
Deceased donor, *n (%)*	73 (95)	22 (92)	29 (94)	22 (100)	0.55
HLA A, B, mismatch, *median (IQR)*	3 (2–4)	3.0 (2.2–4.0)	2.5 (2.0–3.2)	3.0 (2.0–3.7)	0.33
HLA DRB, DQB, mismatch, *median (IQR)*	2 (1–3)	2.0 (1.2–3.0)	2.0 (1.0–3.0)	1.0 (0.0–2.0)	< *0.01*
Cold ischemia time, minutes, *median (IQR)*	932 (708–1158)	869 (634–1094)	957 (773–1120)	1079 (810–1202)	0.53
Warm ischemia time, minutes, *median (IQR)*	50 (42–61)	44 (39–56)	48 (42–65)	56 (50–65)	0.16
Anti-CD25 induction, *n (%)*	32 (42)	5 (21)	16 (52)	11 (50)	*0.04*
Thymoglobulin induction, *n (%)*	43 (56)	19 (79)	15 (48)	9 (41)	*0.02*
**At the time of TMA diagnosis**					
Recipient age, year, median (IQR)	51 (37–67)	48 (33–67)	61 (39–67)	50 (38–66)	0.68
Women, n (%)	31 (40)	9 (38)	12 (39)	10 (44)	0.84
Time post-transplantation, days, median (IQR)	954 (8–2695)	3 (1–8)	1079 (182–2257)	3200 (1777–5646)	-
Previous episode of TMA, n (%)	7 (9)	4 (17)	1 (3)	2 (9)	0.25
History of ABMR, *n (%)*		-	6 (19)	3 (14)	0.71
Intensive care unit hospitalization	14 (18)	2 (8)	8 (26)	4 (18)	0.26
Steroids, *n (%)*	71 (92)	23 (96)	27 (87)	21 (95)	0.36
CNI, *n (%)*	66 (86)	20 (83)	28 (90)	18 (82)	0.71
mTOR inhibitors, *n (%)*	7 (9)	1 (4)	5 (16)	1 (5)	0.32
Mycophenolate mofetil, *n (%)*	65 (84)	22 (92)	26 (84)	17 (77)	0.42
Combination of CNI and mTOR inhibitor, *n (%)*	4 (5)	1 (4)	3 (10)	0 (0)	0.45
Clinical presentation					
Systolic BP, median (IQR)	153 (130–170)	144 (129–168)	148 (130–161)	167 (152–182)	0.44
Diastolic BP, median (IQR)	85 (70–101)	84 (78–100)	79 (65–90)	101 (84–106)	< 0,01
Neurologic symptoms, n (%)	8 (10)	1 (4)	5 (16)	2 (9)	0.37
Diarrhea, n (%)	16 (21)	1 (4)	10 (32)	5 (23)	0.03
Purpura, n (%)	3 (4)	1 (4)	1 (3)	1 (5)	1.00
Fever, n (%)	14 (18)	1 (4)	10 (32)	3 (14)	0.03
Recurrent herpes zoster, n (%)	1 (1)	0 (0)	0 (0)	1 (5)	0.29
Biological presentation					
Anemia, n (%)	76 (99)	24 (100)	30 (97)	22 (100)	1.00
Hemoglobin levels, g/dL, median (IQR)	89 (80–98)	8.9 (8.6–9.5)	9.7 (9.1–10.6)	7.7 (6.1–9.3)	< 0.001
Mean cell volume, per 1 µm3, median (IQR)	89 (84–94)	90 (86–95)	89 (82–93)	88 (86–93)	0.61
Presence of schistocytes, n (%)	57 (74)	17 (71)	23 (74)	17 (77)	0.88
Platelet count < 150.103/µL, n (%)	63 (82)	18 (75)	27 (87)	18 (82)	0.52
Platelet count, 103/µL, median (IQR)	102 (75–141)	95 (73–160)	113 (76–137)	101 (68–139)	0.95
Neutropenia, n (%)	17 (22)	5 (21)	11 (35)	1 (5)	0.03
Lactate dehydrogenase, xUNL, median (IQR)	2 (1.5–2)	2 (1.5–3.0)	2 (1.0–2.0)	2 (2.0–3.0)	0.12
Lactate dehydrogenase, > UNL, n (%)	59 (77)	19 (79)	21 (68)	19 (86)	0.28
Low haptoglobin levels, n (%)	62 (81)	22 (92)	21 (68)	19 (86)	0.07
Fibrinogen, mg/dL, median (IQR)	400 (300–500)	351 (300–586)	375 (296–500)	375 (330–508)	0.81
C-reactive protein > 10 mg/L, n (%)	38 (49)	14 (58)	14 (45)	10 (45)	0.36
Donor specific anti-HLA antibody, n (%)	22 (29)	8 (33)	5 (16)	9 (41)	0.12
Tacrolimus trough level, ng/mL, median (IQR)	8 (5–11)	8.7 (5.1–11.5)	8.0 (6.2–11.9)	4.1 (3.3–7.2)	< 0.01
Tacrolimus trough level > 12 ng/mL, n (%)	9 (12)	3 (13)	6 (19)	0 (0)	0.09
Serum creatinine, µmol/L, median (IQR)	294 (166–510)	266 (137–532)	256 (155–335)	572 (353–783)	< 0.001
Proteinuria, n (%)	64 (83)	23 (96)	23 (74)	18 (86)	0.09
Proteinuria, g/day, median (IQR)	1.6 (0.4–3.4)	1.6 (1.1–3.3)	0.4 (0.2–1.9)	2.5 (1.2–7.0)	< 0.001

*TMA* Thrombotic microangiopathy,* BP* Blood pressure, *HLA* Human Leukocyte Antigen, *UNL* Upper normal limit, *mTOR* Mammalian target of rapamycin, *CNI* Calcineurin inhibitor, *ABMR* Antibody-mediated rejection

The second group of patients (*n* = 31) had a TMA any time after the second week following transplantation (Unexp-TMA): these 31 patients represented 40.2% of all TMA and their incidence was 1.5/1000 patient-years. Median time to TMA was 3.0 (0.5–6.2) years. In these patients, clinical manifestations of TMA wer more frequent, hemoglobin levels was higher (9.7 g/dl [9.1–10.6], *p* < 0.001) and neutropenia was more frequent (35% of cases, *p* = 0.03) vs the other 2 groups. Low-grade proteinuria was frequent, but proteinuria levels were greater vs the other 2 groups (Table 1). History of ABMR was frequent in unexp-TMA (19%). Median tacrolimus trough level was 8.0 ng/mL (6.2–11.9).

The third group of patients (Fail-TMA, *n* = 22) had a TMA in patients with failing grafts: these 22 patients represented 28.6% of all TMA and their incidence was 2.2% of all graft losses. In this group, median time post-transplantation was 8.8 years (4.9–15.5) (Table 1). DSA were found in 41% of patients with Fail-TMA. Systolic blood pressure significantly increased one month before TMA event in fail-TMAs compared to unexp-TMA (*p* < 0.01) (Figure S1). Serum creatinine levels were significantly higher in the 3 months before TMA event in the fail-TMA group compared to the unexp-TMA group (*p* < 0.01) and on the first day of TMA with 572 µmol/L (353–783) and 256 µmol/L (155–335), respectively (*p* < 0.0001). Diastolic blood pressure was higher in the fail-TMA group than eTMA and unexp-TMA groups with 101 mmHg (84–106), 84 mmHg (78–100) and 79 mmHg (65–90), respectively (*p* < 0.01).

### Causes of TMA in the 3 groups of patients

#### Complement alternative pathway and ADAMTS13 exploration

In the 3 groups, most patients had 2 or more intricated causes of TMA (Table 2). The diagnosis work-up was incomplete in many patients: C3/C4 were measured in only 21 (87.5%), 20 (64.5%) and 13 (59.1%) patients, and low C3 was found in 7 (33%), 2 (10%) and 4 (31%) patients, respectively, in eTMA, unexp-TMA and fail-TMA groups. Complement alternative pathway (anti-Factor H antibody) was explored in 6, 7 and 3 patients, respectively, in these 3 groups: only 6 patients had atypical HUS (representing 4%, 10% and 9%, respectively, in these 3 groups). Among these 6 patients, 1 patient had been diagnosed before transplantation and had an early recurrence, one was considered as Alport syndrome, and the last 4 patients had nephropathy of unknow origin. One patient had a hybrid complement factor H receptor 1 (CFHR1)/CFH mutation, another patient had a CFH mutation, and one patient had a CD46 mutation. Chronic active ABMR and ABMR were identified as a trigger for aHUS in 4 patients and no trigger was identified in 2. None of the patients had thrombotic thrombocytopenic purpura (ADAMTS13 activity testing was measured in 6 patients (7.8%) and was always superior to 20%).

**Table 2:** Identified causes or triggers of thrombotic microangiopathies

**Causes**	All	Early TMA	Unexpected TMA	Failure TMA	*P*-value
	*n* = *77*	*n* = 24	*n* = 31	*n* = 22	
** ≥ 2 causes or triggers**	62 (81)	22 (92)	22 (71)	18 (82)	0.15
**Primary TMAs**					
Atypical HUS	6 (8)	1 (4)	3 (10)	2 (9)	0.76
**Secondary TMAs**					
Acute antibody-mediated rejection	12 (16)	4 (17)	4 (13)	4 (18)	1.00
Chronic antibody-mediated rejection	15 (19)	0 (0)	1 (4)	14 (64)	< *0.001*
Shiga toxin *Escherichia coli* HUS	2 (3)	0 (0)	2 (6)	0 (0)	0.33
**Infections**	32 (42)	7 (29)	16 (52)	9 (41)	0.24
Bacteria	17 (22)	6 (25)	6 (19)	5 (23)	0.93
*Escherichia coli* (without evidence of shiga toxin)	4 (5)	3 (13)	0 (0)	1 (5)	0.96
*Other gram-negative bacilli*	7 (9)	1 (4)	3 (10)	3 (14)	0.48
*Streptococcus*	2 (3)	2 (8)	0 (0)	0 (0)	0.17
*Staphylococcus*	1 (1)	0 (0)	0 (0)	1 (5)	0.29
**Virus**	17 (22)	1 (4)	10 (32)	6 (27)	*0.03*
*Epstein-Barr virus*	9 (12)	0 (0)	6 (19)	3 (14)	0.06
*Cytomegalovirus*	9 (12)	0 (0)	5 (16)	4 (18)	0.06
*Other*	2 (3)	1 (4)	1 (3)	0 (0)	1.00
Not documented	3 (4)	1 (4)	2 (6)	0 (0)	0.78
Pre-eclampsia	1 (1)	0 (0)	1 (3)	0 (0)	1.00
Malignant hypertension	8 (10	3 (13)	1 (3)	4 (18)	0.22
Malignancies	9 (12)	1 (4)	2 (6)	6 (27)	*0.04*
Autoimmune diseases	3 (4)	0 (0)	3 (10)	0 (0)	0.11
CNI toxicity, n (%)	6 (8)	2 (8)	4 (13)	0 (0)	0.27
Combination of CNI and mTOR inhibitor, *n (%)*	4 (5)	1 (4)	3 (10)	0 (0)	0.45
Conditions associated with other TMAs	30 (39)	13 (54)	8 (26)	9 (41)	0.01
Large hematoma	9 (12)	4 (17)	4 (13)	1 (5)	0.45
Folate deficiency	19 (25)	8 (33)	3 (10)	8 (36)	*0.04*
Vitamin B12 deficiency	2 (3)	1 (4)	1 (3)	0 (0)	1.00

*TMA* Thrombotic microangiopathy, *HUS* Hemolytic and uremic syndrome, *CNI* Calcineurin inhibitors

*mTOR* Mammalian target of Rapamycin

#### Most frequent causes of TMA in the 3 groups

As shown in Table 2, infections (29%), were the most common cause of TMA among patients with eTMA. Acute antibody-mediated rejection (ABMR) was diagnosed in 4 patients. Of note, folate deficiency was present in 8 patients. Malignant hypertension was present in 13% of eTMA patients.

Infections were the most frequent cause of unexp-TMAs (52%). Viral reactivation (EBV and CMV) was more frequent in unexp-TMA compared to eTMA and fail-TMA with 32%, 0% and 27%, respectively (*p* = 0.03). CNI toxicity based on tacrolimus trough level above 12 ng/mL (8% of TMAs). Other frequent causes of TMA were ABMR (13%), aHUS (10%), auto-immune disease (10%) and shigatoxin-associated HUS (6%).

Among patients with fail-TMA, acute ABMR and chronic active ABMR were present in 18% and 64% of patients, respectively. Infections (41% of fail-TMA patients, with EBV and CMV reactivations in 14 and 18%, respectively), malignancies (27% compared with 4% and 6% in eTMA and unexp-TMA, respectively, *p* = 0.04) and malignant hypertension (18%) were commonly associated with TMA.

#### Histology findings of patients with TMA

Overall, 27 patients (35%) had a kidney biopsy (8 patients, 10 patients and 9 patients in the eTMA, unexp-TMA and fail-TMA groups, respectively) (Table S1). Capillary luminal narrowing, glomerular capillary congestion, double contours and mesangial cell proliferation were the most frequent TMA-associated lesions observed. TMA histologic features were present in 4, 5, and 6 patients in the eTMA, unexp-TMA and fail-TMA, respectively. Microvascular inflammation (MVI) was defined according to Banff classification by the sum of glomerulitis (g) score and peritubular capillaritis (cpt) score over 2 [6]. MVI occurred in 4, 3 and 4 patients in the eTMA, unexp-TMA and fail-TMA, respectively. Double contours were present in 66% of fail-TMA biopsies (6 patients out of 9 who underwent biopsy; 4 patients out of 6 who presented microvascular inflammation with g + cpt ≥ 2). Grade 3 interstitial fibrosis with tubular atrophy was more frequent in the fail-TMA group (*p* < 0.01).

### Management and long-term outcome

#### Early TMAs

CNI were systematically maintained as compared to unexp-TMAs and fail-TMAs, but doses were reduced in 25% of patients. Fourteen patients (58%) required red cell transfusions (Table S2).

Follow-up was in this group was 30 (14–46) months. No MACE was observed. Graft survival was 100% and TMA recurrence was 8% (Table 3). At the last follow-up, serum creatinine level was 145 µmol/L (110–194).

**Table 3:** Long-term outcome after TMA

**Clinical outcomes**	Early TMA	Unexpected TMA	Failure TMA	*P*-value
	*n* = 24	*n* = 31	*n* = 22	
**In-hospital adverse event**				
Death, *n (%)*	0 (0)	0 (0)	3 (14)	< *0.05*
Major cardiovascular event, *n (%)*	0 (0)	0 (0)	3 (14)	< *0.05*
Renal replacement therapy, *n (%)*	2 (8)	3 (10)	22 (100)	-
Graft loss, *n (%)*	0 (0)	0 (0)	22 (100)	*-*
**Clinical outcomes at last follow-up**				
Time to last follow-up, months, *median (IQR)*	30 (14–46)	23 (16–48)	24 (3–58)	0.85
Death, *n (%)*	1 (4)	4 (13)	1 (5)	0.49
Serum creatinine levels, µmol/L, *median (IQR)*	145 (110–194)	193 (100–293)	-	0.26
Graft loss, *n (%)*	0 (0)	6 (19)	-	0.03
TCMR, *n (%)*	1 (4)	3 (10)	-	0.62
ABMR, *n (%)*	2 (8)	4 (13)	-	0.68
Recurrent TMA during follow-up, *n (%)*	2 (8)	5 (16)	6 (27)	0.23
Retransplantation, *n (%)*	0 (0)	1 (3)	10 (45)	*-*

*TMA* Thrombotic microangiopathy, *IS* Immunosuppressive, *TCMR* T cell-mediated rejection, *ABMR* Antibody-mediated rejection

#### Unexp-TMA

Change in immunosuppressive therapy was frequent (58%). Red cell transfusion was less frequently necessary than in other TMA groups with 16%, 58% and 59% in unexp-TMA, eTMA and fail-TMA, respectively (*p* =  < 0.001). Three patients (10%) required temporary renal replacement therapy.

**Fig. 2:**
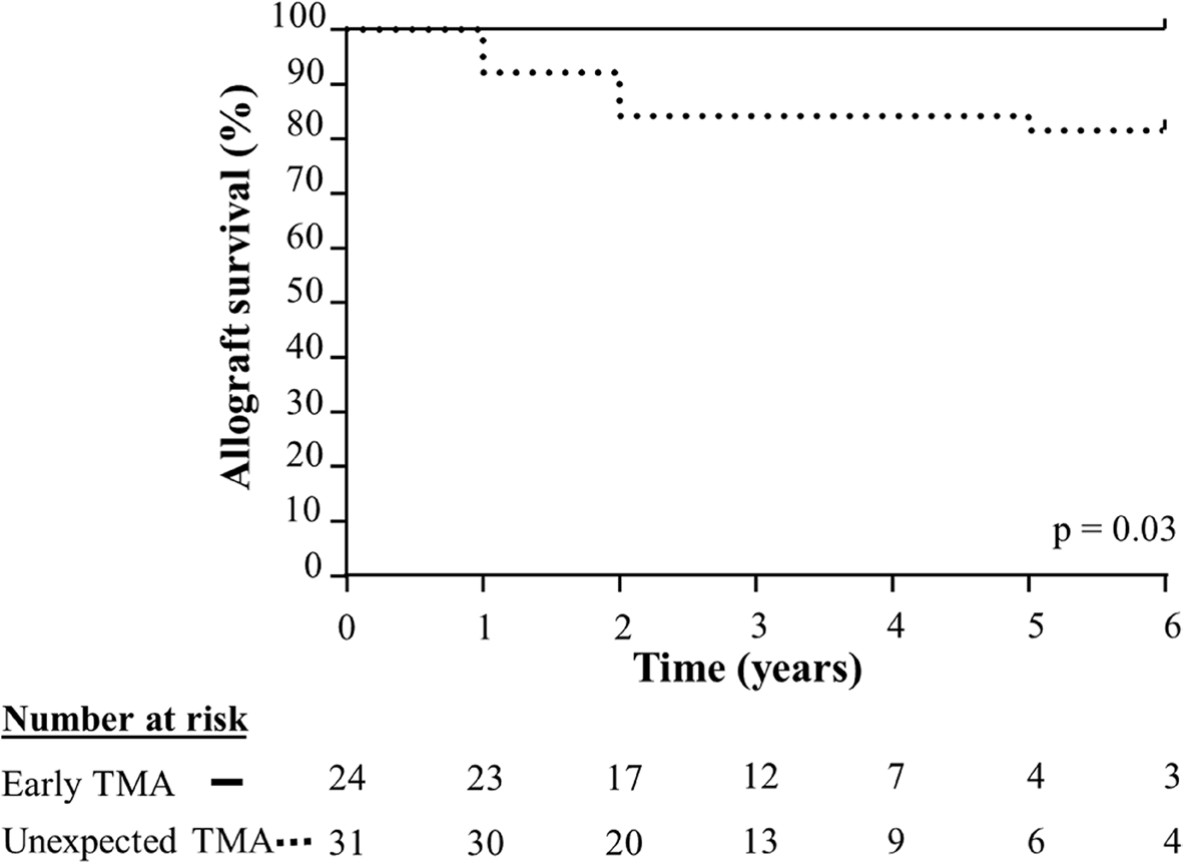
Overall allograft survival during follow-up

Graft survival was 81% with graft loss occurring in 6 patients after a median follow-up of 23 (16–48) months (Fig. 2). No MACE was observed. Recurrence of TMA was observed in 5 patients (16%). At the last follow-up, serum creatinine levels was 193 (100–293) µmol/L.

#### Fail-TMA

Three patients died and 3 patients had a MACE during hospitalisation. Transplantectomy was prompted in 15 patients (68%) due to TMA. Chemotherapy for concomitant cancer was administered in 6 patients (27%).

Graft survival was 0% and 6 patients (27%) had a recurrence of TMA during follow-up (during dialysis after a new kidney transplantation).

## Discussion

In the present study performed in consecutive KTR, the epidemiology of TMA varied according to their TMA timing and 3 periods could be distinguished with distinct incidences, causes and prognosis. Early TMA was present in 1.5% of KTR within the first two weeks following transplantation, unexpected TMA occurred later, any time during follow-up in 1.5 per 1000 patient-years and graft failure-associated TMA accompanied graft failures in 2.2% of patients. Early TMA more frequently observed in the presence of anti-HLA class-II antibodies, preformed DSA and induction with thymoglobulin were associated with acute ABMR and bacterial infections and had an excellent prognosis. Unexpected TMA, more associated with viral (EVM/CMV) than bacterial infections and triggered by acute/chronic active ABMR and mTOR inhibitor-CNI combination even though it was not statistically significant. Poor prognosis graft failure-associated TMA presented with severe/malignant hypertension and heavy proteinuria were triggered by malignancies, acute/chronic ABMR, and infections. No thrombotic thrombocytopenic purpura was observed, typical and atypical HUS and isolated CNI toxicity were rare. When biopsy was performed, features of TMA was present in two third of patients.

First, we observed that secondary TMA constituted 93% of TMA in KTR in modern era. A study from the USRDS was published before the eculizumab era and indicated a 4.9/1000 PY incidence of de novo TMA among 15,870 KTR between 1998–2000, but a 189/1,000 incidence in patients with history of TMA/HUS [4]. Risk factors included younger recipient age, older donor age, female recipient, and initial use of sirolimus [4]. According to Ponticelli and Banfi, “the most important risk factors were cyclosporin and tacrolimus, as well as by anti-mTOR drugs” [13]. Our results demonstrate that considerable progress has occurred since, leading to a complete modification of TMA epidemiology. Interestingly, a recent study from our group indicated that secondary TMA also constitute more than 90% of all TMA in nontransplanted patients [3]. However, the incidence of TMA is much more frequent in KTR than in nontransplanted patients [3], supporting the role of transplantation-specific risk factors (in addition to nonspecific parameters) in the development of TMA.

Second, acute ABMR (15.6%) and chronic active ABMR (19.5%) were important and perhaps underestimated causes of TMA, but other (mostly bacterial infections) triggers were usually present. Of note, acute ABMR was diagnosed in some patients many months before TMA onset, and no further biopsy was performed (explaining discordance between Table S1 and Table 2 regarding humoral-mediated rejections). Acute and chronic lesions were described but due to the limited sample size, it was not possible to assess the prognostic value of acute and chronic lesions. Interestingly, TMA was present in patients with DSA without evidence of rejection. The immediate post-transplant period was characterized by a relatively high (1.5%) incidence of TMA. These results are in concordance with the study of Reynolds et al. indicating that the risk of TMA was highest for the first 3 months after transplant [4]. The median time to TMA was 25 days in the study of Caires et al. [14]. In this early period, risk factors were the presence of HLA class II antibodies, preformed DSA and induction with thymoglobulin, with or without acute ABMR. In the retrospective study of Satoskar et al. in KTR, de novo TMA was present in 6.1% patients who had a biopsy and half of them with C4d positive biopsy [5]. In the study of Ozdemir et al. assessing the risk factors of TMA among selected KTR with acute cellular or humoral rejection, chronic rejection, polyomavirus infection, IgA nephropathy recurrence, acute humoral rejection (33.3%), acute cellular rejection (17.6%) and chronic active humoral rejection (41.5%) were the most frequent causes [15]. However, patients were selected and included based on biopsy findings. Our results stress that acute ABMR and chronic ABMR are important but not the most single causes of TMA. Whether HLA class II antibodies or preformed DSA could promote endothelial damage leading to systemic TMA without acute ABMR or chronic ABMR is unknown and must be addressed. Interestingly, we found that 4 patients had only renal-limited TMA but not hematological TMA. Whether the pathophysiology of renal-limited TMA and TMA with renal and hematological features is identical is possible [16]. Recently, it was shown that HLA class II antibodies could induce necrotic cell death in human endothelial cells [17]. It is acknowledged that chronic ABMR is associated with C4d deposits in peritubular capillaries but whether these antibodies can lead to isolated systemic TMA is unknown. Sreedharanunni et al. showed coexistence of renal TMA and transplant glomerulopathy in 18.4% of biopsies with TMA features but no information on systemic TMA was given [18]. Of note, induction with thymoglobulin was performed in many patients, especially in those with high immunological risk in our study. However, although it was shown that thymoglobulin can bind to endothelium [19], the pathogenic role of thymoglobulin in TMA development is presently unproven.

Third, we found that isolated CNI toxicity is now rare, strongly suggesting that the transplant community has integrated this CNI side effect and closely monitor drug through levels. This is consistent with a frequent 25% decrease of CNI dose in eTMA group and change in immunosuppressive therapy in 58% of unexp-TMAs. This finding is in contrast with the large body of literature stressing the major role of CNI-induced TMA in KTR [20–24]. However, we also found that TMA was present in 4 patients who had a combination of CNI and mTOR inhibitor. Endothelial damage can be observed as a result of CNI use and/or mTOR inhibitors [25]. Ponticelli and Banfi emphasized the role of these drugs in the development of TMA in KTR, especially when these agents were used together [13]. In a recent paper, we underlined the role of the concept of “multiple hits” to explain the high frequency of multiple triggers or causes in patients with TMA [3]. These older data and the present ones support this view and CNI management is crucial.

Fourth, we observed that bacterial infections (17/77, 21.0%) as well as viral infections (17/77, 21.0%) were the single most frequent associated factors or triggers of TMA in KTR. Enterobacteria were the most frequent identified bacteria (similar epidemiology in observed in nontransplanted populations) [26]. Bacterial translocation from infectious sites could cause endothelial damage and von Willebrand factor release [27–29] with or without complement activation leading to TMA development. CMV and other viruses could also have a pathogenic role in TMA [30] but the relationship with EBV seems unclear.

Five, graft survival was excellent in patients early TMA, less good in patients with unexpected TMA and very poor in patients with failing kidneys. In the latter situation, patients usually presented with severe/malignant hypertension. It is presently unknown whether severely elevated blood pressure is evidence of extensive parenchymal damage associated with TMA or may play a role in the development of TMA in transplanted and nontransplanted populations [3, 31–34].

Our study has limitations. It is a retrospective study; however, the retrospective nature of this study probably has not impact as selection bias seems unlikely (all patients followed in our center were analyzed, all consecutive TMA patients were included). The true prevalence may be underestimated, but it is unlikely since our patients are seen in our ward at least every year until graft loss or death. It is a real-life study: diagnostic work-up (especially those related to exploration of the complement activation pathway) was not performed in all patients, and management was heterogeneous. A complement genetic panel is recommended for all patients with transplant-associated TMA, even in the absence of TMA before transplantation as complement-mediated TMA seem to be responsible in most recurrent cases [35] and up 30% of the de novo cases [36]. It can be argued that TMA is a pathologic diagnosis, and that only 27/77 patients had a kidney biopsy. However, two-third of patients with biopsy showed features of renal TMA, and kidney biopsy is usually avoided as TMA is identified as a risk factor of bleeding [37, 38]. More histology studies are required to assess the diagnostic value of glomerular and arteriolar lesions in patients with TMA [39].

Our study has also some strength. All consecutive patients were included reducing selection bias. Its strength also derives from the careful review of all individual files (not administrative codes). The diagnosis of TMA was adjudicated by three physicians familiar with the disease. The incidence of primary and secondary was estimated in an unbiased fashion. The nosology that was proposed allows identification of patients with distinct presentation, risk factors and prognosis.

In conclusion, this study constitutes one of the first large-scale clinical pictures of TMA in KTR. It gives a clear estimation of the frequency and incidence of distinct types of TMA according to duration of follow-up. Our findings highlight the major role of infections and allo-immunization in the development of TMA and underline that the very low rate of isolated CNI toxicity and aHUS in modern era is probably due to the progress in management of immunosuppressive drugs and of aHUS in KTR.

### Supplementary Information

**Additional file 1:** **Figure S1.** Blood pressure and serum creatinine levels preceding TMA.** Table S1.** Renal allograft biopsies at the time of TMA. **Table S2.** Therapeutic management of TMAs.

## Data Availability

The datasets used and/or analyzed during the current study are available from the corresponding author on reasonable request.

## Abbreviations

aHUS: Atypical haemolytic and uremic syndrome
ABMR: Antibody-mediated rejection
BP: Blood pressure
CFHR: Complement factor H receptor
CMV: Cytomegalovirus
CNI: Calcineurin inhibitor
Cpt: Peritubular capillaritis
D0: Day zero
DSA: Donor-specific antibody
EBV: Epstein-Barr virus
eTMA: Early TMA
Fail-TMA: Graft failure-associated TMA
g: Glomerulitis
HLA: Human leucocyte antigen
IS: Immunosuppressive
KTR: Kidney transplant recipients
LDH: Lactate dehydrogenase
MACE: Major adverse cardiovascular events
MVI: Microvascular inflammation
mTOR: Mammalian target of rapamycin
RRT: Renal Replacement therapy
TCMR: T cell-mediated rejection
TMA: Thrombotic microangiopathies
TTP: Thrombotic thrombocytopenic purpura
Unexp-TMA: Unexpected TMA
UNL: Upper Normal limit
