# Supplementary material for: Thrombotic microangiopathies after kidney transplantation in modern era: nosology based on chronology
Source: BMC Nephrol. 2023 Sep 20;24:278. doi: 10.1186/s12882-023-03326-8 (PMC10512637; doi:10.1186/s12882-023-03326-8)
Supplement: Supplementary file 1 — Additional file 1: Figure S1. Blood pressure and serum creatinine levels preceding TMA. Table S1. Renal allograft biopsies at the time of TMA. Table S2. Therapeutic management of TMAs. [file 12882_2023_3326_MOESM1_ESM.docx]

**Supplementary data**

## **Figure S1: Blood pressure and serum creatinine levels preceding TMA**

Diastolic BP was significantly higher in terminal TMAs compared to intermediate TMAs. Patients with terminal TMA had worse renal function. TMA: thrombotic microangiopathies; BP: blood pressure; s: systolic; d: diastolic, ns : not significant, * : p=0.05, ** : p=0.01, *** : p =0.001, ****: p=0.0001.

## Figure S1: Blood pressure and serum creatinine levels preceding TMA


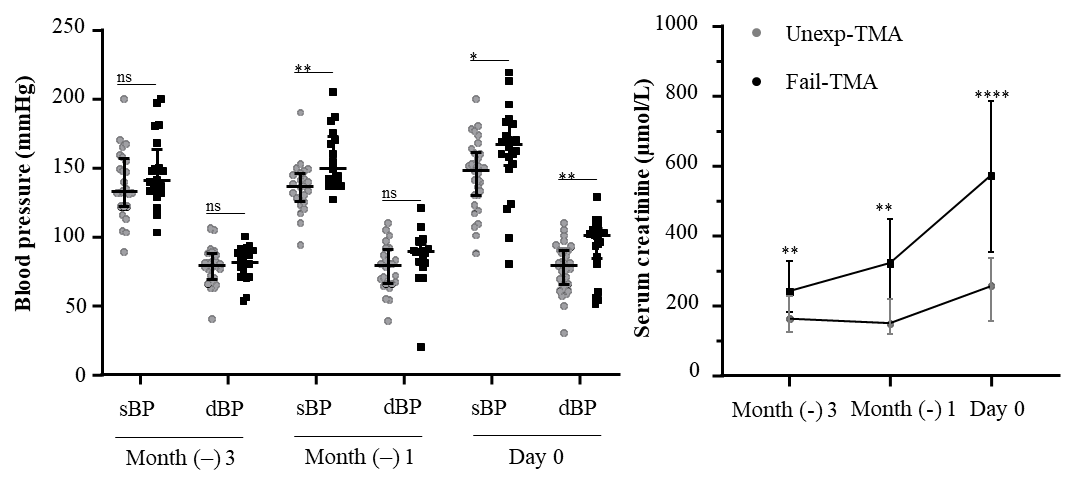


## Table S1: Renal allograft biopsies at the time of TMA

TMA: thrombotic microangiopathies (e: early; i: intermediate; t: terminal). t: tubulitis; i: interstitial inflammation; g: glomerulitis; v: intimal arteritis; cg: GBM double contours; cpt: peritubular capillaritis; ci: interstitial fibrosis; ct: tubular atrophy; cv: vascular fibrous intimal thickening.

| **Patients** | **TOTAL GLOMERULI** | **ENDOTHELIAL SWELLING** | **CAPILLARY LUMINAL NARROWING** | **DOUBLE CONTOURS** | **MESANGIOLYSIS** | **FIBRIN DEPOSIT IN CAPILLARY LUMINA** | **CAPILLARY INTRALUMINAL THROMBI** | **GLOMERULAR CAPILLARY CONGESTION** | **MESANGIAL CELL PROLIFERATION** | **CAPILLARY COLLAPSE** | **GLOMERULAR NECROSIS** | **C4d** | **t** | **i** | **g** | **v** | **cg** | **cpt** | **ci** | **ct** | **cv** | **g+cpt** |
| --- | --- | --- | --- | --- | --- | --- | --- | --- | --- | --- | --- | --- | --- | --- | --- | --- | --- | --- | --- | --- | --- | --- |
| **Early TMA** |  |  |  |  |  |  |  |  |  |  |  |  |  |  |  |  |  |  |  |  |  |  |
| Patient 1 | 20 | no | yes | no | no | no | yes | no | no | no | no | 0 | 0 | 1 | 1 | 1 | 0 | 2 | 2 | 2 | 1 | 3 |
| Patient 2 | 7 | no | no | no | no | no | no | no | no | no | no | 0 | - | - | - | 1 | - | - | 3 | 3 | - | - |
| Patient 3 | 15 | no | no | no | no | no | no | no | no | no | no | 0 | 0 | 0 | 1 | 0 | 0 | 1 | 1 | 1 | 3 | 2 |
| Patient 4 | 20 | no | no | yes | no | no | yes | yes | no | no | no | 0 | 0 | 0 | 1 | 0 | 1 | 0 | 1 | 1 | 2 | 1 |
| Patient 5 | 15 | no | no | no | no | no | no | no | no | no | no | 0 | 0 | 0 | 0 | 0 | 0 | 0 | 1 | 1 | 1 | 0 |
| Patient 6 | 20 | no | no | no | no | no | yes | no | no | no | no | 0 | 0 | 0 | 2 | 0 | 0 | 0 | 0 | 0 | 0 | 2 |
| Patient 7 | 16 | no | yes | no | no | yes | no | yes | no | no | no | 1 | 1 | 0 | 1 | 0 | 0 | 2 | 0 | 0 | 1 | 3 |
| Patient 8 | 15 | yes | yes | no | no | no | no | yes | no | no | no | 0 | 3 | 3 | 0 | 0 | 0 | 1 | 0 | 0 | 0 | 1 |
| **Unexpected TMA** |  |  |  |  |  |  |  |  |  |  |  |  |  |  |  |  |  |  |  |  |  |  |
| Patient 9 | 30 | yes | no | no | no | no | no | no | no | no | no | 0 | 1 | 0 | 0 | 1 | 0 | 0 | 1 | 2 | 1 | 0 |
| Patient 10 | 5 | no | no | no | no | no | no | no | no | no | no | 1 | 0 | 0 | 3 | 1 | 0 | 3 | 0 | 0 | 0 | 6 |
| Patient 11 | 22 | no | no | no | no | no | no | no | yes | no | no | 0 | 0 | 0 | 1 | 0 | 0 | 0 | 0 | 0 | 0 | 1 |
| Patient 11 | 25 | no | no | no | no | no | yes | yes | yes | no | yes | 0 | 0 | 0 | 1 | 0 | 0 | 0 | 1 | 1 | 0 | 1 |
| Patient 13 | 20 | no | no | no | no | no | no | no | yes | no | no | 0 | 0 | 0 | 0 | 0 | 0 | 0 | 2 | 2 | 0 | 0 |
| Patient 14 | 30 | no | no | no | no | no | no | no | no | yes | no | 0 | 0 | 0 | 0 | 0 | 0 | 0 | 1 | 1 | 1 | 0 |
| Patient 15 | 44 | no | yes | yes | yes | no | yes | yes | no | no | yes | 0 | 0 | 0 | 1 | 0 | 1 | 0 | 3 | 3 | 1 | 1 |
| Patient 16 | 17 | no | no | no | yes | no | no | no | no | no | no | 0 | 1 | 0 | 2 | 0 | 0 | 2 | 3 | 3 | 3 | 4 |
| Patient 17 | 15 | no | no | no | yes | no | no | no | no | no | no | 0 | 1 | 0 | 1 | 0 | 0 | 0 | 2 | 2 | 0 | 1 |
| Patient 18 | 15 | no | yes | no | no | yes | no | yes | no | no | no | 1 | 0 | 0 | 3 | 0 | 3 | 2 | 2 | 2 | 2 | 5 |
| **Failure TMA** |  |  |  |  |  |  |  |  |  |  |  |  |  |  |  |  |  |  |  |  |  |  |
| Patient 19 | - | yes | no | yes | no | no | no | no | no | no | no | 1 | - | - | - | - | 3 | 2 | - | - | - | 2 |
| Patient 20 | 40 | no | yes | yes | no | no | no | no | no | no | no | 0 | 1 | 2 | 3 | 2 | 3 | 3 | 3 | 3 | 3 | 6 |
| Patient 21 | 20 | no | no | yes | no | yes | no | no | yes | no | no | 0 | 0 | 0 | 1 | 0 | 2 | - | 3 | 3 | 2 | 1 |
| Patient 22 | 15 | no | no | no | no | no | no | no | yes | no | no | 0 | 0 | 2 | - | 0 | - | - | 3 | 3 | 2 | - |
| Patient 23 | 20 | no | yes | no | no | no | no | yes | yes | no | no | 0 | 0 | 0 | - | 0 | - | - | 3 | 3 | 2 | - |
| Patient 24 | - | no | no | yes | no | no | no | no | no | no | no | 1 | 2 | 1 | 3 | 0 | 3 | - | 2 | 2 | 2 | 3 |
| Patient 25 | 13 | no | yes | yes | no | no | no | no | no | no | no | 0 | 3 | 0 | - | 0 | - | 1 | 3 | 3 | 3 | 1 |
| Patient 26 | 30 | no | yes | no | yes | no | yes | no | no | no | no | 0 | 0 | 0 | 1 | 0 | 3 | 0 | 3 | 3 | 2 | 1 |
| Patient 27 | 7 | no | no | no | no | no | no | yes | no | no | no | 0 | 0 | 0 | 2 | 0 | 3 | 2 | 3 | 3 | 3 | 4 |

## Table S2: Therapeutic management of TMAs

Chi2 test or Fisher test for qualitative data and Kruskal Wallis test for quantitative data were used.

CNI: calcineurin inhibitors; mTOR inhibitor: mammalian target of rapamycin inhibitor; IV: intravenous.

| **Therapeutic management** | Early TMA | Unexpected TMA | Failure TMA | P-value |
| --- | --- | --- | --- | --- |
|  | *n*=24 | *n*=31 | *n*=22 |  |
| Change in immunosuppressive therapy, *n (%)* | 8 (33) | 18 (58) | 14 (64) | 0.08 |
| Discontinuation of CNI, *n (%)* | 0 (0) | 5 (16) | 6 (27) | *0.01* |
| Lower dose of CNI, *n (%)* | 6 (25) | 7 (23) | 5 (23) | 1.00 |
| Switch of CNI, *n (%)* | 1 (4) | 2 (6) | 1 (5) | 1.00 |
| Discontinuation of mTOR inhibitor, *n (%)* | 0 (0) | 1 (3) | 0 (0) | 1.00 |
| Transplantectomy, *n (%)* | 0 (0) | 0 (0) | 15 (68) | *<0.001* |
| Plasma exchange, *n (%)* | 2 (8) | 3 (10) | 1 (5) | 0.87 |
| Plasma infusion, *n (%)* | 2 (8) | 1 (3) | 2 (9) | 0.61 |
| Eculizumab, *n (%)* | 1 (4) | 2 (6) | 0 (0) | 0.78 |
| Rituximab, *n (%)* | 0 (0) | 2 (6) | 0 (0) | 0.33 |
| Steroid, *n (%)* | 16 (67) | 12 (39) | 13 (59) | 0.10 |
| IV immunoglobulin, *n (%)* | 4 (17) | 4 (13) | 2 (9) | 0.88 |
| Antibiotics/antivirals, *n (%)* | 6 (25) | 13 (42) | 6 (27) | 0.34 |
| Chemotherapy, *n (%)* | 0 (0) | 1 (3) | 6 (27) | *<0.01* |
| Red cell transfusion, *n (%)* | 14 (58) | 5 (16) | 13 (59) | *<0.001* |
| Platelet transfusion, *n (%)* | 0 (0) | 0 (0) | 1 (5) | 0.58 |
